# Supplementary material for: Comparison between USPIOs and SPIOs for Multimodal Imaging of Extracellular Vesicles Extracted from Adipose Tissue-Derived Adult Stem Cells
Source: Int J Mol Sci. 2024 Sep 7;25(17):9701. doi: 10.3390/ijms25179701 (PMC11395141; doi:10.3390/ijms25179701)
Supplement: Supplementary file 1 [file ijms-25-09701-s001.zip › ijms-3160911-supplementary.pdf]

Supplementary Materials

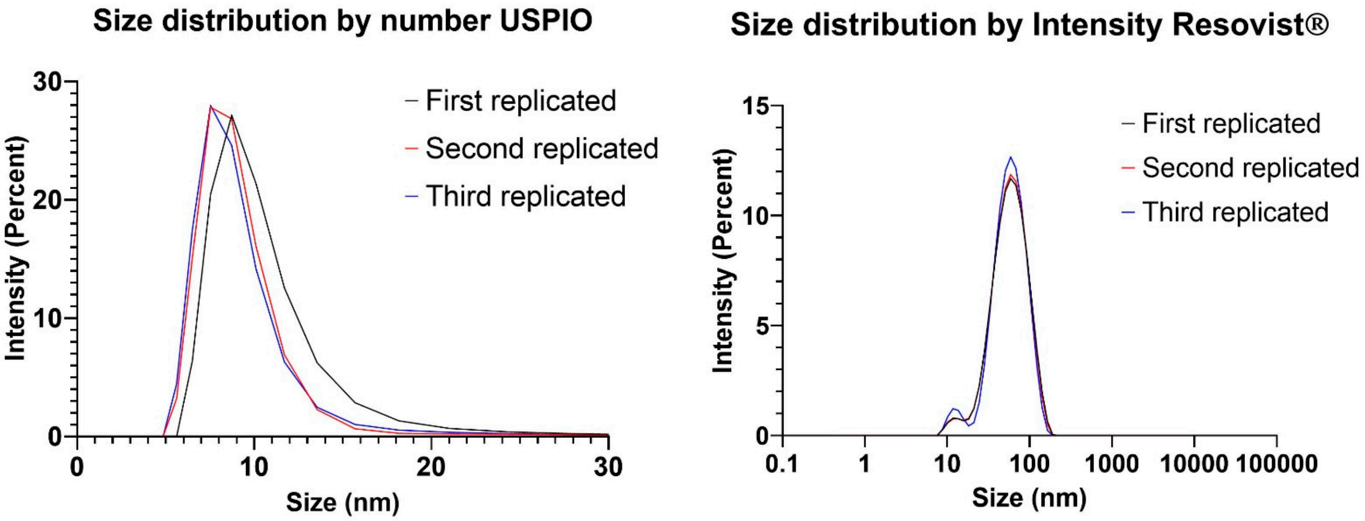

Figure S1. Nanoparticles size characterization determined by DLS. Each nanoparticle was measured in replicate. Due to small size of USPIO, we measured the diameter of nanoparticles analysing the number distribution by DLS.

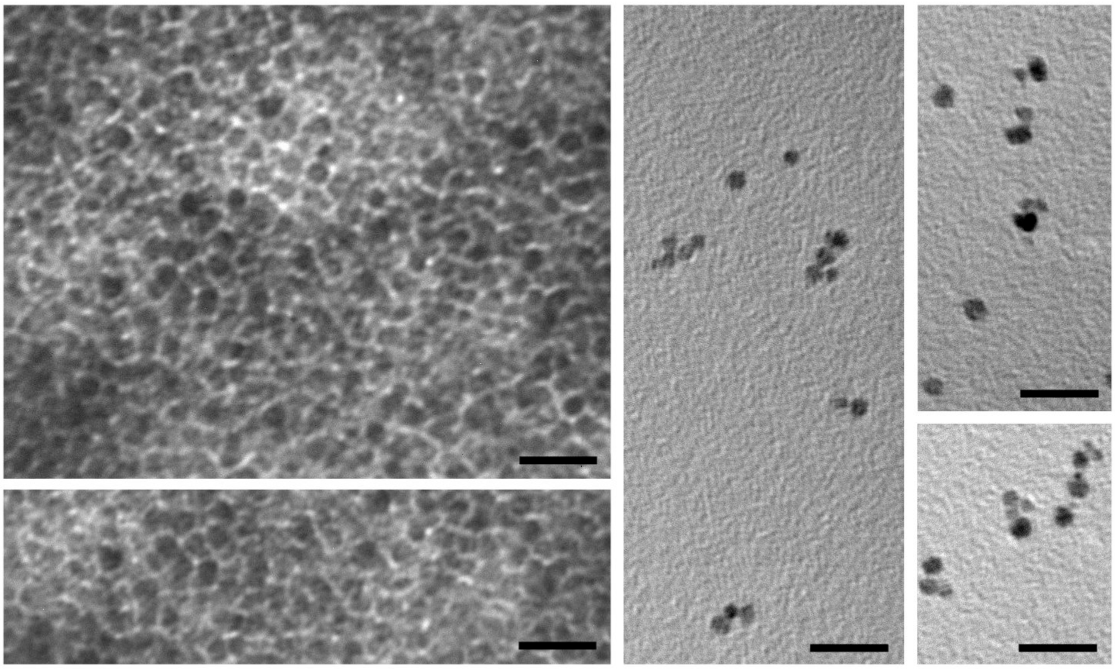

Figure S2. Transmission electron micrographs of USPIO showing a homogeneous distribution of iron oxide nanoparticles. Scale bars: 20 nm

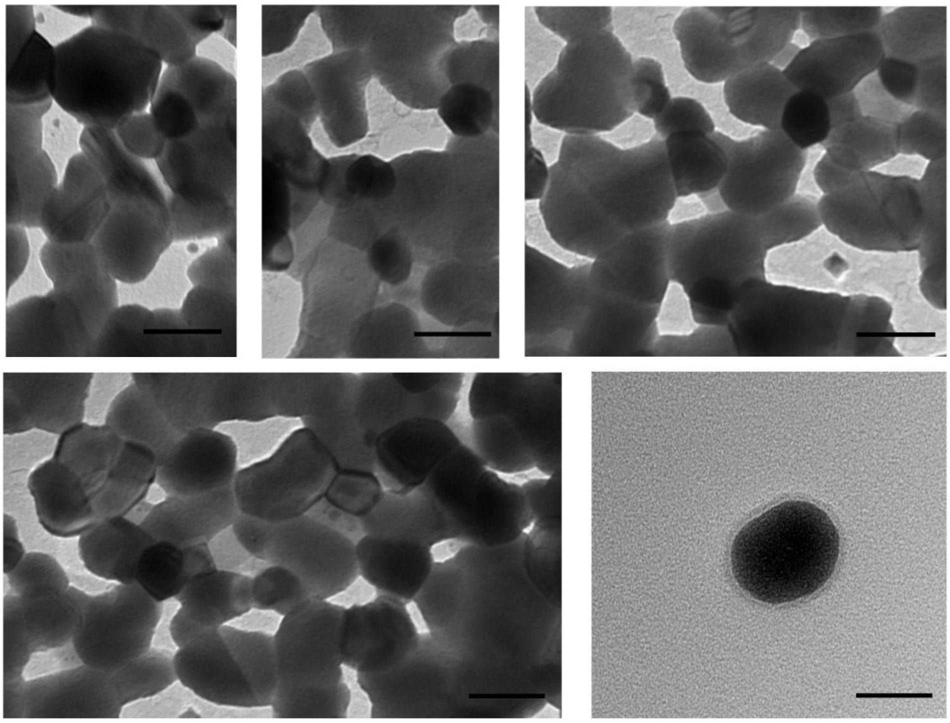

Figure S3. Transmission electron micrographs of Resovist® showing uniform size. Scale bars: 100 nm

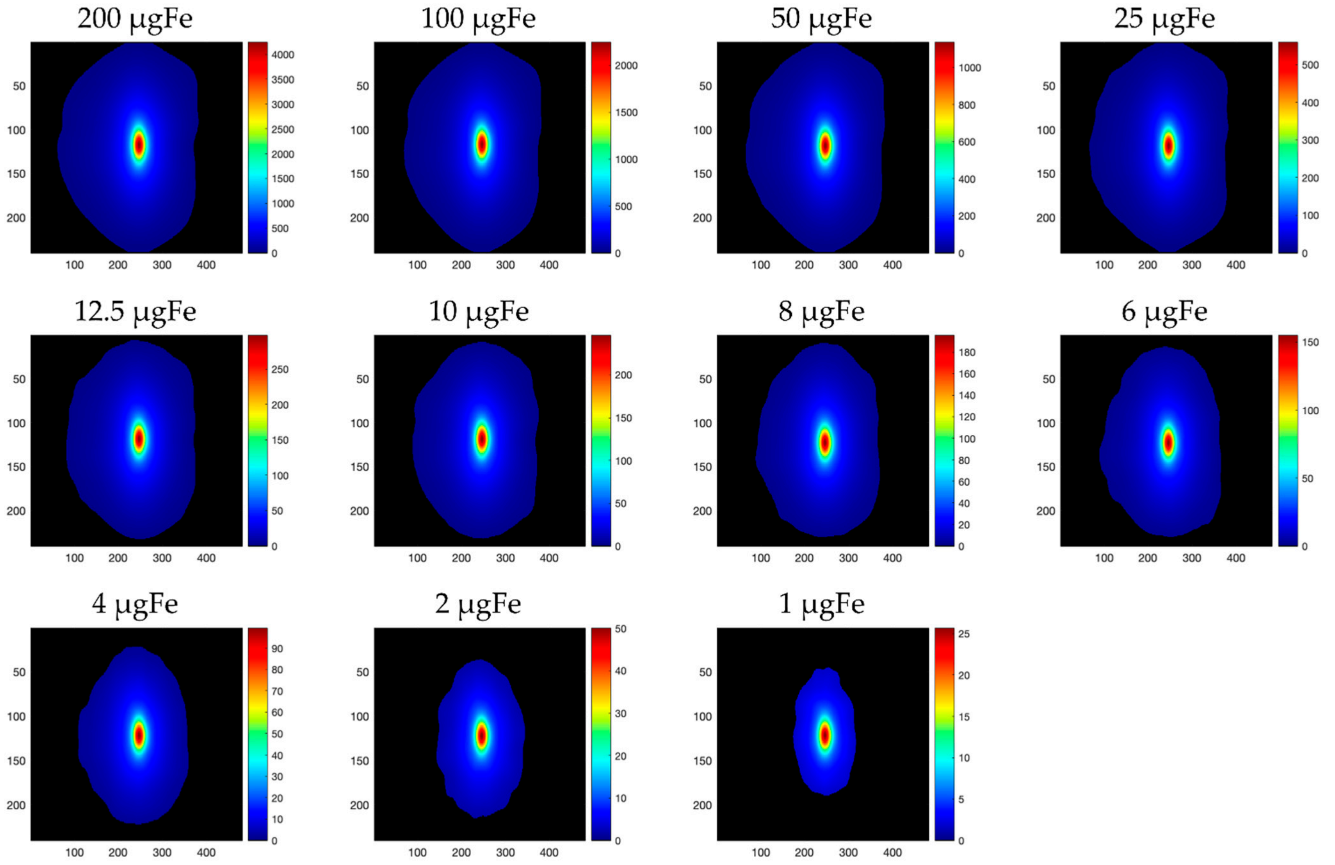

Figure S4. MPI images of decreasing Resovist® concentrations in 150 μL of water at a field strength of 3.055 T/m, with each image normalized to its respective maximum intensity signal.
